# Advancements in Cardiac Amyloidosis Treatment

**DOI:** 10.3390/biomedicines13010079

**Published:** 2024-12-31

**Authors:** Tarek Ziad Arabi, Abdullah Shaik, Ahmed El-Shaer, Omar Al Tamimi, Eman Nayaz Ahmed, Mohamad S. Alabdaljabar, Ahmad Safdar, Ali Mushtaq

**Affiliations:** 1 College of Medicine, Alfaisal University, Riyadh 11533, Saudi Arabia; 2 Department of Internal Medicine, Henry Ford St. John Hospital, Detroit, MI 48236, USA; 3 Department of Internal Medicine, Creighton University School of Medicine, Omaha, NE 68124, USA; 4 Internal Medicine, Southern Illinois University School of Medicine, Springfield, IL 62702, USA; 5 Department of Internal Medicine, Mayo Clinic, Rochester, MN 55905, USA; 6 Department of Internal Medicine, Cleveland Clinic Foundation, Cleveland, OH 44195, USA

**Keywords:** cardiac amyloidosis, treatment, diagnosis, challenges

## Abstract

Cardiac amyloidosis (CA) is a progressive condition resulting from the deposition of amyloid fibrils in the heart, which leads to severe diastolic dysfunction and restrictive cardiomyopathy. The disease has two main subtypes: light-chain and transthyretin (TTR) CA, with the latter subdivided into wild-type and hereditary forms. Despite advances in diagnostic imaging, early detection remains a challenge due to non-specific symptoms that mimic other cardiac conditions. Treatment has evolved significantly with targeted therapies like TTR stabilizers, gene silencers, and RNA interference, showing promise in altering disease progression. However, barriers such as high costs, limited availability of genetic testing, and inadequate multidisciplinary care continue to impede comprehensive management. Future strategies should focus on integrating novel gene-editing therapies, expanding access to diagnostics, and enhancing multidisciplinary care models to improve outcomes. Overall, early diagnosis, equitable access to therapies, and personalized management plans are crucial to advancing care for CA patients.

## 1. Introduction

Amyloidosis is described as the systemic pathologic deposition of misfolded proteins that leads to the aggregation and formation of amyloid fibrils that are resistant to degradation and that are observed in multiple organs such as the heart, kidneys, liver, gastrointestinal tract, and peripheral nervous system [1,2,3]. Cardiac amyloidosis (CA) is characterized by the deposition, aggregation, and infiltration of extracellular amyloid fibrils and misfolded proteins into the myocardium. The hallmark histologic finding is green birefringence under polarized light following Congo red staining [4,5]. There are more than 36 known proteins that can aggregate as amyloids; however, only a few have been documented to cause overt cardiac conditions [5,6].

CA stems most commonly from the immunoglobulin light chain (monoclonal AL or primary amyloidosis) and transthyretin (ATTR). Transthyretin-CA can be caused by either normal transthyretin protein misfolding due to aging and is known as wild-type ATTR (ATTR-wt), previously termed senile or age-related CA. Another cause of ATTR is known as variant ATTR (ATTR-v), which is due to misfolding from abnormal genetic sequences, previously known as familial CA [7]. The prevalence of ATTR cardiac amyloidosis (ATTR-CA) is higher in males, particularly among older patients with the wild-type form and those with hereditary variants, such as the Val122Ile mutation [8,9].

## 2. CA Etiologies

CA induces cardiac dysfunction through the progressive deposition of amyloid within the myocardium, leading to stiffening, myocardial thickening, restrictive physiology, and diastolic dysfunction [4,10]. Furthermore, CA is associated with atrial and ventricular arrhythmias stemming from electromechanical changes as a result of amyloid deposition [11]. Although CA leads to restrictive cardiomyopathy with diastolic dysfunction, the degree and stage of presentation varies with the type of amyloidosis (Table 1). In AL-CA, it can occur early with little or no wall thickening, while ATTR-CA typically develops restrictive dysfunction in the mid-to-late stages [12]. The overall prevalence of CA is difficult to assess, as a high degree of suspicion is required for its diagnosis due to the variable causes and insights into the clinical signs of CA; however, CA is classified as a rare disorder (i.e., a condition affecting fewer than 5 individuals out of 10,000). Other reports estimate the prevalence of AL-CA to be 18% among all CA patients [13] and that of ATTR-wt CA to be 13% of all patients hospitalized with heart failure (HF) with preserved ejection fraction (HFpEF) [14,15]. ATTR-v CA demonstrates a higher prevalence in certain populations due to its genetic predisposition. Jacobson et al. report that the Val122Ile mutation, a common form of ATTR-v CA, is seen in 3–4% of the African–Caribbean population [16]. With the advances in diagnosing modalities, ATTR-wt CA may very well become the most commonly diagnosed form of CA [2].

## 3. Advancements in CA Diagnosis

Delays in diagnosis remain a significant barrier in CA care, as non-specific symptoms such as fatigue, dyspnea, and peripheral edema often mimic conditions like HFpEF. Early diagnosis of CA, irrespective of subtype, is an important predictor of outcomes [17,23]. Differentiating between AL and ATTR amyloidosis is critical due to their distinct management pathways, but this is often hindered by limited access to advanced diagnostic modalities such as cardiac magnetic resonance (CMR), bone scintigraphy, and liquid chromatography–tandem mass spectrometry.

Historically, CA was diagnosed by endomyocardial biopsy or through an extracardiac biopsy positive for amyloid with a left ventricle (LV) wall thickness >12 mm upon echocardiography without other explainable causes [5,23]. Biopsy offers high accuracy for diagnosing CA; however, it does not quantify the extent of amyloid infiltration in the heart. Moreover, the diagnostic yield of biopsies is also contingent upon the site of the biopsy and the specific amyloid variant. Hence, non-invasive diagnostic modalities, particularly imaging techniques, have become the preferred initial approach for diagnosing CA [24].

ATTR CA can be diagnosed without histological findings on biopsy if the following criteria are met: heart failure with echocardiogram or CMR findings suggestive of amyloidosis; grade 2 or 3 cardiac uptake on radionuclide scan of 99mTc-PYP, 99mTc-HMDP, or 99mTc-DPD; and the absence of detectable monoclonal gammopathy (immunofixation; serum/urine protein electrophoresis, serum/urine immunofixation, and free light chain assays) [25,26,27].

With the advent of imaging modalities, different imaging tests provide differing insights into the pathogenesis and functional deficits of amyloid deposition; hence, patients typically undergo many tests for a complete evaluation. The common imaging modalities for CA are echocardiography, CMR, and radionuclide imaging [28].

### 3.1. Echocardiography

Echocardiography is the most commonly utilized non-invasive modality of choice to assess cardiac structure and function, and it is also the preferred initial diagnostic tool for investigating cardiac symptoms when CA is suspected [29].

LV hypertrophy (LVH) is the most common finding on echocardiography, as the ventricular wall is the preferred site of amyloid deposition [23,29,30]. Notably, the pattern of LVH varies by the type of CA. AL-CA typically presents with symmetrical concentric remodeling, whereas ATTR-CA is more commonly associated with asymmetrical LVH [29]. In the absence of aortic valve disease or severe hypertension, an LV thickness of >12 mm and grade II diastolic dysfunction raise concerns about CA. However, one-third of CA patients may have normal LV thickness, meaning that LVH does not always correlate with amyloid deposition, and CA cannot be ruled out in the absence of LVH [30,31,32]. CA is not only limited to the LV, but amyloid deposits can also affect the right ventricle (RV), atria, interatrial and interventricular septums. The RV wall thickness may exceed 5 mm, and the atrial septum (>5 mm) or atrioventricular valve (>2 mm) may thicken if the atria are involved [33,34]. With bilateral ventricular hypertrophy and concomitant diastolic dysfunction, increased ventricular pressure is the sequela leading to increased bilateral atrial pressure and enlargement [30]. Rahman et al. found that a combination of low voltage upon electrocardiogram (ECG) and increased myocardial thickness had a sensitivity of 72% and a specificity of 91% for diagnosing cardiac amyloidosis [35]. Increased LV wall thickness, particularly when associated with a granular or speckled myocardial appearance, is a hallmark of CA. This finding has a sensitivity of 87% and a specificity of 81% for diagnosing CA [18]. Enlarged left atria can also illustrate the severity and duration of diastolic cardiac dysfunction which serves as marker for early changes in ATTR-CA [36]. Interestingly, an atrial septal thickness of over 6 mm is 100% specific for diagnosing CA, albeit in only a small subset of patients [30,34]. Clinically, there are some poor echocardiographic signs in CA patients, which include pericardial/pleural effusion, thick valves, a thick interatrial septum, low stroke volume, paradoxical low gradient in aortic stenosis, restrictive LV filling, and decreased global longitudinal strain (GLS) upon strain analysis [23]. GLS is a measure of the longitudinal shortening of the myocardium during the cardiac cycle and is typically reduced in CA due to amyloid infiltration.

There is still a need for increased awareness, expertise, and clinical suspicion, as initial ECG and echocardiography findings may not be typical [29]. Although echocardiography has been the primary mode of imaging and assessment, it is always utilized along with clinical presentation, biomarkers (troponin, N-terminal pro-B-type natriuretic peptide (NT-proBNP)), and ECG. In summary, typical cardiac echo features for CA that are seen in patients are an LV wall thickness ≥ 12 mm, ≥ grade 2 diastolic dysfunction, and reduced Doppler velocities [37,38].

### 3.2. Cardiac Magnetic Resonance (CMR)

Over the past decade, CMR has established itself as a powerful imaging modality. Its utilization has grown due to its noninvasive nature, and the superior accuracy and precision it provides compared with echocardiography while also offering insight into the presence, distribution, and extent of hypertrophy [39]. CMR enhances diagnostic accuracy through various techniques, including late gadolinium enhancement (LGE), GLS with apical sparing, T1 mapping with pre- and post-contrast, and extracellular volume (ECV) imaging [23].

LGE employs gadolinium agents and is a key imaging technique for diagnosing CA through “nulling”, a process of adjusting the inversion recovery time so that the normal myocardium appears black or “nulled” [40]. If “nulling” the myocardium is challenging, this is highly suggestive of CA, as this technique highlights abnormal tissue, such as areas affected by disease, which retain the gadolinium contrast and appear brighter, making it easier to detect cardiac amyloid infiltration [23]. Phase-sensitive inversion recovery has improved LGE imaging, showing global subendocardial or transmural enhancement with high sensitivity and specificity while reducing operator dependency [41]. However, LGE cannot differentiate CA subtypes and is difficult to quantify, making T1 mapping a valuable complementary tool for earlier detection and for monitoring disease progression [23].

T1 refers to the longitudinal relaxation time during CMR and measures how quickly the heart tissue realigns with the magnetic field. In CA, elevated T1 values indicate amyloid deposition or fibrosis. Novel CMR tissue characterization techniques, such as native T1 mapping (without the use of contrast agents), help overcome the limitations of traditional imaging methods [42,43]. Studies have demonstrated the diagnostic potential of native T1 mapping in detecting suspected AL cardiac amyloidosis, offering a valuable tool for identifying the disease, even in early stages [44].

Additionally, CMR enables the quantification of amyloid burden using T1 mapping and ECV measurement [44,45]. T1 mapping performed after gadolinium administration allows for the calculation of the ECV fraction, which is notably elevated in cases of CA infiltration, being greater than or equal to 0.40, and it also serves as an indication of severity [23,45]. ECV measurements, although pathognomonic in patients with biopsy-proven amyloidosis, are not specific for amyloidosis and can be elevated in other forms of cardiovascular disease, including reactive or replacement fibrosis and inflammation [46]. Hence, when using CMR to assess CA, the criteria for diagnosis are an LV wall thickness ≥ 12 mm, global ECV > 0.40, diffuse LGE enhancement, and abnormal gadolinium kinetics [5,38].

Similar to echocardiography, CMR-derived GLS is significantly impaired compared with that in healthy controls and in patients with other cardiomyopathies. A characteristic finding in CA is the pattern of “apical sparing”, where the apical segments show relatively preserved strain compared with the basal and mid-ventricular segments, sometimes referred to as the “cherry on top”. This pattern is highly suggestive of CA and helps differentiate it from other causes of LVH [47].

### 3.3. Bone Tracer Scintigraphy with Bone SPECT Radiotracer

Myocardial uptake of Technetium-labeled isotopes has long been linked with CA in bone scintigraphy imaging, with the leading three identified isotopes being 99mTc-pyrophosphate (99mTc-PYP), 99mTc-3,3-diphosphono-1,2-propanodicarboxylic acid (99mTc-DPD), and 99mTc-hydroxymethylene diphosphonate (HMDP), with 99mTc-PYP the only agent available in the United States [38].

99mTc-PYP scintigraphy essentially involves the administration of 10–25 mCi of radiotracer, which is subsequently followed by planar and SPECT imaging at 1 h or 3 h post-injection [38,48]. These images are visually graded on a bone scan by qualitative and quantitative scores (Table 2), where the qualitative scores compare the heart uptake with that of rib/bone, while the quantitative scores rely on the heart-to-contralateral (H/CL) ratio for 99mTc-PYP, where the amount of tracer absorbed by the heart is compared with that at the opposite side of the chest [38,48]. A visual score of 2 or 3 on planar imaging, or a heart-to-contralateral chest (H/CL) ratio of 1.5 or higher, strongly indicates the presence of ATTR-CA. The 99mTc-PYP scan has shown a sensitivity of 94% and a specificity of 89% for diagnosing ATTR-CA, with a specificity of 100% for grade 3 scans [49].

False positives were primarily seen in patients with AL amyloidosis as it is noted to have, at times, low-grade uptake with clonal immunoglobulins or light-chain abnormalities, highlighting the need for serum/urine testing to exclude these before interpreting scintigraphy results [26]. Gilmore et al. confirmed that in patients without monoclonal gammopathy, grade 2 or higher myocardial uptake was highly specific for an ATTR-CM diagnosis [26].

### 3.4. Biomarkers

The utility of biomarkers, such as troponin and NT-proBNP, has also been studied for the diagnosis of CA. Vergaro et al. conducted a multicenter European study to evaluate the diagnostic utility of the biomarkers in CA [51]. The study identified optimal biomarker cut-offs for ruling out and ruling in CA. Values of NT-proBNP < 180 ng/L and high-sensitivity troponin-T (hs-TnT) < 14 ng/L demonstrate high sensitivity, effectively excluding the diagnosis of CA with minimal false negatives. On the other hand, hs-TnT ≥ 86 ng/L showed high specificity, providing a reliable threshold for confirming CA. Importantly, combining these biomarkers further improved the diagnostic precision, particularly when both were below the rule-out thresholds. The results were also validated across diverse cohorts, including in suspected AL-CA cases and in patients with increased left ventricular wall thickness, demonstrating robust performance. However, reduced diagnostic accuracy was observed in patients with severe renal impairment or atrial fibrillation.

Takashio et al. also evaluated the diagnostic utility of hs-TnT in differentiating CA from other causes of cardiac hypertrophy [52]. Their study demonstrated that hs-cTnT levels were significantly elevated in CA patients compared with those with non-amyloid hypertrophy, with a cut-off value of 0.0312 ng/mL achieving a sensitivity of 74% and a specificity of 76%. Among the subtypes, hs-TnT levels were highest in AL-CA compared with ATTRwt-CA and ATTR-v CA. Furthermore, hs-TnT outperformed other biomarkers such as BNP and echocardiographic measures like the E/e’ ratio in diagnostic accuracy. Multivariate analysis also identified hs-TnT as an independent diagnostic marker, highlighting its utility in clinical settings.

Another study by Kumar et al. developed a revised staging system for AL-amyloidosis that improves patient risk stratification by combining cardiac biomarkers (NT-proBNP ≥ 1800 pg/mL and troponin-T ≥ 0.025 ng/mL) with serum-free light chain (FLC-diff ≥ 18 mg/dL) [53]. Patients were grouped into four stages, with the median overall survival decreasing from 94.1 months in stage I to 5.8 months in stage IV. Overall, it is evident that cardiac biomarkers have proven to be useful in the diagnosis of CA, especially when used in adjunct with other traditional diagnostic methods.

Finally, artificial intelligence (AI) has begun taking a prominent role in the diagnosis of CA. For example, an AI-enhanced 6-lead ECG model demonstrated an area under the curve (AUC) of 0.9 and a precision of 0.85 [54]. Supporting these findings, a recent meta-analysis found that AI-enhanced ECGs had a pooled AUC of 0.89, 0.9, and 0.8 for CA, ATTR-CA, and CA-AL, respectively [55]. Other models have also been developed for echocardiograms [56] and 99^m^Tc-scintigraphy [57] with promising results. Although further studies are needed, it appears that AI will play a larger role in CA diagnosis in the future.

## 4. Treatment of CA

Managing CA-related cardiac complications is uniquely challenging due to the severe diastolic dysfunction and restrictive physiology inherent to the disease. Additionally, there remains a significant unmet need for therapies that target the removal of existing amyloid deposits, as current approaches focus primarily on slowing the disease progression. Furthermore, toxicity and tolerability issues with targeted interventions, such as tafamidis, can limit their applicability, particularly in advanced disease stages (e.g., New York Heart Association (NYHA) class IV patients).

Since cardiac amyloidosis can arise from different types of amyloid deposits, treatment strategies vary based on the underlying cause. For ATTR-CA, treatment focuses on transthyretin tetramer stabilizers, which prevent TTR dissociation, TTR silencers that inhibit synthesis of transthyretin, and TTR disruptors that help clear existing amyloid deposits. In contrast, the primary goal of AL-CA is to eradicate those abnormal plasma cells in circulation that produce these toxic light chains.

### 4.1. An Overview of Convential CA Therapies

#### Overview of Conventional ATTR-CA Therapies

Earlier studies that reported a lack of efficacy or poor tolerability of HF medications in ATTR-CA were largely conducted in patients with advanced cardiac disease. However, more recently, diagnoses occurring at an earlier stage have allowed for a better evaluation of these treatment options.

A recent analysis from the National Amyloidosis Centre, involving over 2000 patients, has provided the first evidence that conventional HF medications in ATTR-CA, specifically mineralocorticoid receptor antagonists (MRAs) and beta-blockers (BBs), may offer therapeutic benefits for patients with ATTR-CA [58]. The study demonstrated that treatment with MRAs was linked to a reduction in mortality across the entire patient population, particularly in those with a left ventricular ejection fraction (LVEF) > 40%. However, there was no significant benefit for patients with an LVEF ≤ 40%. This is an important consideration, as it is contrary to what is implemented for HF patients and may suggest that goal-directed medical therapy (GDMT) may be more effective in the early stages of cardiac amyloidosis [58]. On the other hand, low-dose BBs were associated with reduced mortality only in patients with an LVEF of 40% or less. This was observed even after excluding patients with ischemic heart disease, reinforcing the established role of BBs in HFrEF by mitigating harmful adrenergic and neurohormonal activation [58]. However, the meta-analysis by Kang et al. showed no significant link between BBs and all-cause mortality, suggesting that although BBs are frequently prescribed, their effectiveness in improving survival ATTR-CA is still unclear [59].

Furthermore, the usage of sodium-glucose cotransporter-2 inhibitors (SGLT2is) is being evaluated as an option for CA treatment. In a propensity score-matched study, SGLT2i treatment was associated with a lower risk of HF-related hospitalization and cardiovascular and all-cause mortality [60]. However, these results are from observational data sets, and prospective randomized trials are necessary to validate the safety and efficacy of these treatments in this particularly vulnerable patient population [61].

### 4.2. Targeted Therapies for ATTR-CA

#### 4.2.1. TTR Stabilizers

Tafamidis, a selective TTR stabilizer, binds to the thyroxine-binding sites of the TTR tetramer, slowing its dissociation into monomers—a key step in amyloid formation [62]. This action helps to slow disease progression in both cardiomyopathy and peripheral neuropathy [63]. Currently, tafamidis is the only U.S. Food and Drug Administration (FDA)-approved therapy for ATTR-CM with a Class I, level B-R recommendation for treating patients with either ATTRwt-CA or ATTRv-CA and NYHA class I-III symptoms [64]. Selecting the appropriate patient population is crucial, as tafamidis has not demonstrated benefits for individuals with NYHA class IV symptoms, severe aortic stenosis, or significantly reduced renal function (eGFR < 25 mL/min/1.73 m^2^) [65]. Data from the ATTR-ACT trial and its long-term extension also suggest improved survival in NYHA class III patients continuously treated with tafamidis compared with those on a placebo [66,67]; however, further research is needed to clarify its role in advanced disease. Additionally, the recent ATTRibute-CM trial results for another TTR stabilizer, acoramidis, have shown a significantly superior primary hierarchical outcome, incorporating measures of mortality, morbidity, and functional status compared with the placebo [68].

#### 4.2.2. TTR Gene Silencers

These new therapies have recently emerged as promising therapies for CA, particularly for slowing the progression of amyloidosis-related polyneuropathy in ATTRv. These drugs work by addressing the underlying cause of TTR amyloidosis, lowering the production of misfolded TTR protein and potentially slowing disease progression [69]. Patisiran is a small double-stranded interfering RNA encapsulated in a lipid nanoparticle [70,71]. Through distinct mechanisms, patisiran therapy promotes the degradation of TTR mRNA in hepatocytes, leading to a reduction of serum TTR levels by approximately 75–90% compared with pre-treatment values. A subgroup analysis of the APOLLO trial showed that ATTR–polyneuropathy patients with cardiomyopathy who received patisiran experienced improvements in their myocardial structure, lower NT-proBNP levels, and a faster gait speed compared with the placebo [72]. The APOLLO-B study further demonstrated patisiran’s efficacy in wild-type and variant ATTR-CM patients, showing slower declines in the 6-min-walk test distance and Kansas City Cardiomyopathy Questionnaire (KCCQ) scores over 12 months, with sustained benefits observed in the 18-month open-label extension phase [73]. However, the FDA concluded that patisiran’s effects on ATTR-CM were not clinically significant enough to approve its expanded use for cardiomyopathy. Recent data from the ongoing HELIOS-B trial, a double-blind, randomized clinical study evaluating the second-generation siRNA vutrisiran, have shown encouraging outcomes for ATTR-CA patients [74]. Preliminary data from the HELIOS-B trial, showed that vutrisiran had a 28% reduction in the composite of all-cause mortality and recurrent cardiovascular events compared with the placebo [74]. Meanwhile, the NEURO-TTRansform trial showed that eplontersen treatment is associated with improvements in LVEF and stroke volume in patients with cardiomyopathy at 65 weeks [75]. The value of second-generation antisense oligonucleotides is currently under investigation in randomized clinical trials of eplontersen in patients with ATTR-CA (ClinicalTrials.gov registration ID NCT04136171).

#### 4.2.3. Antibody-Mediated Removal of ATTR-CA

TTR tetramer stabilizers, silencers, and gene-editing therapies have been developed to prevent ATTR buildup; however, these treatments do not directly address the amyloid already deposited in the heart [76]. Amyloid deposits elicit a minimal response in tissues, and amyloid fibrils are known to be highly stable and resistant to enzymatic breakdown in vitro [77]. A phase 1 double-blind, placebo-controlled randomized trial was conducted to evaluate a recombinant human IgG1 antibody, NI006, designed to target TTR, in 40 patients with ATTR-CA [78]. Early signs of efficacy were encouraging and included reductions in serum cardiac biomarkers, decreased cardiac uptake of bone tracers, and lower myocardial extracellular volume values, all of which suggest a potential reduction in the CA burden [78]. Further support for the potential therapeutic role of anti-amyloid antibodies comes from studies involving three unique patients with ATTR-CA who experienced unprecedented spontaneous recovery [79]. These patients demonstrated near-complete clearance of their CA deposits and were found to have circulating polyclonal IgG antibodies that specifically targeted ATTR. Remarkably, amyloid clearance was also associated with significant cardiac remodeling, with both structure and function improving toward normal levels without evidence of fibrosis. These findings strongly suggest that ATTR-CA may be a reversible condition, offering hope even for those with advanced stages of the disease.

#### 4.2.4. Novel CRISPR-Cas9 Gene-Editing Therapy

CRISPR-Cas9 gene editing is an innovative technology that allows for the precise modification of specific genes, making it a promising tool for treating genetic diseases at their source [80,81]. In ATTR-CA, CRISPR-Cas9 has been utilized to target and reduce the production of TTR. NTLA-2001, a CRISPR-Cas9-based therapy delivered through lipid nanoparticles, specifically targets liver cells, where TTR is synthesized [82]. In a phase I trial, single doses of NTLA-2001 led to substantial dose-dependent reductions in serum TTR levels, with a 52% mean reduction at the 0.1 mg/kg dose and 87% at 0.3 mg/kg by day 28 [83]. This approach offers the potential for a one-time treatment to significantly decrease the amyloidogenic substrate, potentially slowing or even reversing disease progression in ATTR-CA.

### 4.3. Targeted Therapies for AL-CA

In contemporary clinical practice, the management of AL-CA is built on three established pillars: early disease detection, plasma cell dyscrasia therapies, and supportive measures for end-organ dysfunction [84]. Early disease detection and advances in diagnostics, as discussed earlier, are crucial for timely intervention. In the management of AL-CA, plasma cell dyscrasia therapy should be viewed as “source control”, with the primary objective being the elimination of clonal plasma cells producing the pathogenic light chains, thereby preventing further amyloid deposition and allowing for potential organ recovery [85].

Survival rates for patients with systemic amyloidosis have improved over the past four decades, likely due to early diagnosis and advances in anti-plasma therapies [86,87]. Improvements in hematological and cardiac responses have been interpreted as improvements in survival for patients with CA [88]. One notable advancement is the introduction of daratumumab, especially in combination with cyclophosphamide, bortezomib, and dexamethasone (Dara-CyBorD), which has become a cornerstone treatment for newly diagnosed AL amyloidosis [89]. This demonstrated that the Dara-CyBorD regimen significantly improved cardiac response rates (41.5% vs. 22.2% in the control group) and overall survival, with a hazard ratio of 0.58, in AL-CA patients. Achieving deeper organ responses, particularly in cardiac, renal, and hepatic systems, was associated with a better prognosis. However, the trial excluded patients with advanced Mayo stage III cardiac disease, limiting its applicability to this high-risk group.

Advanced-stage CA, namely, Mayo stages III and IV, is associated with poor survival rates [90]. Survival in advanced heart disease remains a challenge that may be addressed with new novel therapeutics [90]. One proposed strategy to address this has been the development of therapies that can dissolve already deposited fibrils to reverse the disease [91]. A critical fourth pillar of AL-CA management—a disease-modifying approach—is currently missing, as there are no FDA-approved therapies targeting free light chains or existing amyloid deposits [84]. However, two investigational therapies—birtamimab and CAEL-101—may serve as the foundation for this essential missing pillar and help advance its overall management [84].

Birtamimab, previously known as NEOD001, is a monoclonal antibody designed to bind both the soluble light-chain amyloid fibrils (fibrils to be deposited) and the insoluble fibrils that have already been deposited. It neutralizes the soluble fibrils and enhances macrophage-mediated phagocytosis of the existing insoluble amyloid deposits [92]. The VITAL trial was a phase 3 study evaluating the efficacy and safety of birtamimab for patients with AL amyloidosis where patients were randomized to receive either birtamimab plus the standard of care or a placebo plus the standard of care. The trial was halted early as no significant difference was found in the overall mortality or cardiac hospitalization. However, a post hoc analysis conducted by Gertz et al. demonstrated a substantial improvement in mortality for patients with Mayo stage IV AL amyloidosis and comparable tolerability to that of the control arm [91]. As a result, the ongoing confirmatory phase 3 trial, AFFIRM-AL, began with hopes of offering more reliable data for assessing the efficacy and safety of birtamimab in stage IV amyloidosis [91]. The second investigational therapy is CAEL-101, which is a monoclonal antibody derived from the murine monoclonal antibody 11-1F4 that is designed to bind to the insoluble amyloid and evoke antibody-mediated phagocytosis, dissolving misfolded amyloid phase Ia/b light chains [93,94]. In a phase 2 study, CAEL-101 was added to standard therapy (cyclophosphamide–bortezomib–dexamethasone (CyBorD) with or without daratumumab) and demonstrated a tolerable safety profile [93,95]. Currently, two phase 3 clinical trials (NCT04504825 and NCT04512235) are taking place to assess the benefits and safety of incorporating CAEL-01 with CyBorD +/− subcutaneous daratumumab in European Modification Mayo Stages IIIa and IIIb AL-CA.

## 5. Challenges and Barriers to CA Treatment

CA faces several challenges, ranging from delays in diagnosis to difficulties in treatment and patient management. Timely diagnosis is one of the most significant hurdles due to its often subtle and non-specific clinical presentation, including symptoms like fatigue, dyspnea, and peripheral edema, which mimic other heart failure conditions such as HFpEF [96]. This overlap often results in misdiagnosis or late diagnosis, leading to irreversible cardiac damage by the time of proper identification [96,97]. Additionally, overlaps in clinical features with other cardiomyopathies, as well as unexplained LVH and low voltage upon ECG, contribute to diagnostic delays [97,98]. Limited access to liquid chromatography–tandem mass spectrometry and advanced imaging modalities, such as CMR and bone scintigraphy, exacerbates this issue, particularly in resource-limited settings. These modalities are critical for differentiating CA subtypes—AL-CA and ATTR-CA—which have different management approaches and prognoses.

Treatment barriers are equally significant, as standard HF therapies are often ineffective in CA due to its restrictive cardiomyopathy nature, which is characterized by severe diastolic dysfunction and low cardiac output [96]. The effectiveness of treatments like diuretics, BB, and ACE inhibitors is limited, with patients often requiring specialized interventions such as TTR stabilizers, gene silencers, or chemotherapy for AL-CA [96,97]. However, the high cost and limited availability of these targeted therapies restrict their use, especially in low- and middle-income countries [97]. Additionally, regulatory challenges and slow approval processes for new drugs further impede timely access for patients [98]. Drug development itself is complex, as CA involves intricate amyloidogenesis processes, including misfolded protein aggregation, organ infiltration, and cellular toxicity, requiring multi-targeted approaches [98]. Effective CA management is further complicated by the lack of integrated multidisciplinary care. Collaboration among cardiology, hematology, and neurology experts is essential, but such care models are often unavailable, leading to fragmented patient management. Expanding access to genetic testing for hereditary ATTR variants is also imperative, as routine screening can facilitate earlier detection and intervention, benefiting both patients and their at-risk family members.

Clinical trials for novel CA therapies face several barriers, including low patient recruitment due to the disease’s rarity and phenotypic diversity. Achieving robust endpoints also remains a significant challenge, as CA’s variable progression and responses to therapy complicate the establishment of standardized measures of success [96]. Innovative trial designs and enhanced recruitment strategies are needed to address these limitations.

Genetic testing for hereditary ATTR variants is another critical barrier, as it is not uniformly implemented despite its importance in identifying familial cases and guiding treatment decisions [96,97]. A lack of routine genetic screening contributes to missed opportunities for early detection in patients and their at-risk family members [97]. Additionally, a lack of multidisciplinary care is a significant challenge in CA management. Effective treatment requires coordination among cardiology, hematology, and neurology experts, but such integrated care is often unavailable, further complicating patient outcomes [96,98].

## 6. Conclusions

CA remains a challenging condition characterized by delayed diagnosis, complex treatment needs, and significant patient management barriers. Despite recent advancements in diagnostic imaging, genetic screening, and targeted therapies, there is still a critical need for increased awareness among healthcare providers and for the integration of advanced diagnostic tools into routine clinical practice. Improved access to non-invasive diagnostic methods, such as CMR and bone scintigraphy, alongside genetic testing, can facilitate early detection, leading to better outcomes. The development of novel therapeutics, including gene-editing technologies, RNA interference therapies, and next-generation TTR stabilizers, offers hope for more effective and personalized treatments. However, broader efforts are needed to ensure equitable access to these therapies, especially in resource-limited settings. Moving forward, a multidisciplinary approach that includes cardiologists, hematologists, neurologists, and genetic counselors will be essential for comprehensive care. Overall, while progress has been made, a sustained effort is necessary to overcome the persistent challenges in the diagnosis, treatment, and long-term management of cardiac amyloidosis.

## Figures and Tables

**Table 1:** Typical presenting features of subtypes of cardiac amyloidosis.

Feature	AL-CA [5,17,18,19,20]	ATTR-wt CA [5,7,17,18,21]	ATTR-v CA [5,7,18,22]
Age at diagnosis	50+	70+	40+
Gender	Roughly equal	Marked male predominance	Male predominance
Pathophysiology	Disorder of plasma cells	Misfolding of ATTR protein due to biological aging	Misfolding of ATTR protein due to genetic aberration
Involved protein	Immunoglobulin light chain	Transthyretin	Transthyretin
Genetic cause	None	None	Autosomal dominant inheritance; mild male predominance
Extracardiac involvement	Multiorgan involvement	Carpal tunnel, lumbar spine, gastrointestinal tract	Neurologic symptoms (e.g., pain, numbness, tingling)
NTproBNP	Significantly elevated	Elevated	Elevated
Prognosis	~Median survival of up to 13 months (largely variable depending on the cohort)	~Median survival of 75 months	~Median survival of 70 months

**Table 2:** Qualitative assessments of CA that are frequently used in clinical practice.

Grade	Perugini et al. [50]	Dorbala et al. [33]
0	No cardiac uptake, with normal bone uptake	No cardiac uptake, with normal bone uptake
1	Mild cardiac uptake, less than bone uptake	Cardiac uptake less than rib uptake
2	Moderate cardiac uptake with reduced bone uptake	Cardiac uptake equal to rib uptake
3	Strong cardiac uptake with minimal or no bone uptake	Cardiac uptake greater than rib uptake, with minimal or no rib uptake

## Data Availability

No original data were generated in this study.
